# Arboviruses emerging in Brazil: challenges for clinic and implications for public health

**DOI:** 10.1590/S1518-8787.2017051006889

**Published:** 2017-03-29

**Authors:** Maria Rita Donalisio, André Ricardo Ribas Freitas, Andrea Paula Bruno Von Zuben

**Affiliations:** I Departamento de Saúde Coletiva. Faculdade de Ciências Médicas. Universidade Estadual de Campinas. Campinas, SP, Brasil; II Programa Municipal de Controle de Arboviroses. Departamento de Vigilância em Saúde. Secretaria Municipal de Saúde de Campinas. Campinas, SP, Brasil; III Faculdade de Medicina São Leopoldo Mandic. Campinas, SP, Brasil

**Keywords:** Dengue, Zika Virus Infection, Chikungunya Fever, Arbovirus Infections, epidemiology, Communicable Diseases, Emerging

## Abstract

Arboviruses have been emerging in different parts of the world due to genetic changes in the virus, alteration of the host and vector population dynamics, or because of anthropogenic environmental factors. These viruses’ capacity for adaptation is notable, as well as the likelihood of their emergence and establishment in new geographic areas. In Brazilian epidemiologic scenario, the most common arboviruses are DENV, CHIKV, and ZIKV, although others may spread in the country. Little is yet known of the impact of viral co-circulation, which would theoretically result in more intense viremia or other immunological alterations that could trigger autoimmune diseases, such as Guillain-Barré syndrome. The impact on morbidity and mortality intensifies as extensive epidemics lead to a high number of affected individuals, severe cases, and implications for health services, mainly due to the absence of treatment, vaccines, and effective prevention and control measures.

## RESUMO

Notifica-se a emergência de arboviroses em diferentes regiões do planeta em decorrência de mudanças genéticas no vírus, alteração da dinâmica populacional de hospedeiros e vetores ou por fatores ambientais de origem antropogênica. É notável a capacidade de adaptação desses vírus e a possibilidade de emergirem e se estabelecerem em novas áreas geográficas. No contexto epidemiológico brasileiro, os arbovírus de maior circulação são DENV, CHIKV e ZIKV, embora existam outros com potencial de disseminação no País. O impacto da cocirculação viral ainda é pouco conhecido, a qual teoricamente resultaria em viremias mais intensas ou outras alterações imunológicas que poderiam ser o gatilho para doenças autoimunes, como a síndrome de Guillain-Barré. O impacto na morbidade e mortalidade se intensifica à medida que extensas epidemias pressupõem grande número de indivíduos acometidos, casos graves e implicações sobre os serviços de saúde, principalmente diante da ausência de tratamento, vacinas e medidas efetivas de prevenção e controle.

## EMERGENCE OF ARBOVIRUSES

Arboviruses (ARthropod-Borne VIRUS) have caused much concern in public health worldwide. This set is composed of hundreds of viruses that share the characteristic of transmission by arthropods – mostly hematophagous mosquitoes –, although they are not necessarily phylogenetically related^26^. The most important viruses for human health are those transmitted by culicidae, mainly of the *Culex* and *Aedes* genera, although a number of arboviruses can be transmitted by other arthropods, such as sand flies and ticks^26^.

Most arboviruses belong to the *Alphavirus* (*Togaviridae* family) and *Flavivirus* (*Flaviviridae* family) genera; other members important to human health are from the *Bunyaviridae*, *Reoviridae*, and *Rhabdoviridae* families^26^.

This group of RNA viruses represents major genetic plasticity and high mutation frequency, which allows them to adapt to vertebrate and invertebrate hosts^3^. Arboviruses, in general, circulate among wild animals, with some specificity for hosts, maintaining enzootic cycles in few vertebrate and invertebrate species. Humans and pets are generally accidental hosts^14^. This occurs in the circulation of yellow fever, which is found in Brazil in wild outbreaks, without cyclical characteristics, associated with epizooties. We noted the geographic expansion of the circulation of the yellow fever virus from 2000 to 2009^24^ and reemergence in the Midwest and Southeast, since 2014. Another example of enzootic cycles involves the Mayaro virus (MAYV), mainly transmitted by wild mosquitoes of the *Haemagogus* genus, for which the vertebrate hosts are mammal. In humans, it causes fever, headache, exanthema, and arthralgia; however, cases of sustained transmission were not observed. There is evidence of the adaptive capacity of MAYV to alternative cycles involving birds and humans^14^.

The West Nile Virus (WNV) can cause epidemics even in urban areas, as occurs in the United States. It is transmitted by mosquitoes of the *Culex* genus, and its main host is birds^26^. Some viruses lose the demand for enzootic amplification and produce urban epidemics, with humans as exclusive vertebrate amplifiers. Such is the case of the Dengue virus (DENV), Chikunguya (CHIKV), and, lately, Zika (ZIKV)^26^.

The emergent lineage of yellow fever virus in the South region in 2008^24^ is worth noting, with the participation of *Haemagogus leucocelaenus* as the main vector and *Aedes serratus* for transmission^2^. The presence of this last vector in woods near urban areas also in the Southeast of Brazil signals potential for the occurrence of peri-urban cycles of yellow fever^2^. In the current Brazilian epidemiologic scenario, the most common arboviruses are DENV, CHIKV, and ZIKV, as well as the yellow fever virus currently expanding and other arboviruses with potential for dissemination in the country^14^.

The dramatic dissemination of dengue in the Americas in the last decades has been well documented, with over two million notified cases in 2015 (until December 8th), adding up to 1.5 million in Brazil, with 811 deaths and an incidence rate of 763 per 100 thousand inhabitants^a^.

Another forthcoming arbovirus is CHIKV, which began pandemic expansion in 2004. A mutation in an African lineage of CHIKV allowed it to adapt well to the *A. albopictus* vector through the alteration of a protein in the viral envelope E1 (E1-A226V), which was followed by other adaptive steps. This increased the ability of CHIKV to infect and spread in that vector, an abundant species in the Indian Ocean islands and in other regions of Asia^3^. The adaptation favored the expansion of the virus in urban and peri-urban areas on that continent and increased the risk of epidemics in other tropical, subtropical, and even temperate regions, such as Europe. Essa adaptação favoreceu a expansão da virose em áreas urbanas e periurbanas naquele continente e aumentou o risco de epidemias em outras regiões tropicais, subtropicais e mesmo temperadas, como Europa^23^. The autochthonous transmission of an Asian lineage of CHIKV without these mutations was registered in the Caribbean since the end of 2013^13^. In Brazil, autochthonous transmission was detected in September 2014 in Amapá, spreading to other Brazilian states^1^ ^,^ ^21^.

The ZIKV, identified for the first time in Uganda in 1947, had its first documented outbreak only in 2007 in Micronesia. Since then, the transmission area has spread to islands in the Pacific Ocean, especially during a great epidemic in Polynesia in October 2013^5^ ^,^ ^18^. Since April 2015, autochthonous transmission of ZIKV was confirmed in Bahia^1^ and then in Rio Grande do Norte, Pernambuco, Rio de Janeiro, São Paulo, and other states, with patients presenting clinical conditions of exanthematous fever^a^. In the following months, the transmission of ZIKV was confirmed in various countries of the Americas – Colombia in October; Guatemala, El Salvador and Surinam in November; Honduras, Panama, Venezuela, Mexico, and Paraguay in December – where transmission was likely associated with the vector *A. aegypti*. Genetic changes among viral lineages allowed the virus to better adapt to the vector and to human transmission^8^.

The co-circulation of infection by DENV, CHIKV, and ZIKV in Brazil makes clinical management difficult due to similarities; it has implications in the transmission in older people, pregnant women, and young children, and presents a still limited laboratorial rear-guard. Little is yet known of the impact of the co-circulation of this virus. As in the case of reinfection by different serotypes of DENV, the interaction of arboviruses (DENV serotypes 1-4, CHIKV, and ZIKV) could theoretically result in more intense viremia or other immunological alterations which, in turn, would trigger autoimmune diseases such as Guillain-Barré syndrome^4^ ^,^ ^5^ ^,^ ^12^ ^,^ ^18^.

Other arboviruses of the *Flaviviridae* family pose a real threat of epidemic circulation in Brazil, including WNV, the most common arbovirus in the world. Since its introduction in 1999, this virus has rapidly expanded throughout the Americas. In the first decade of the 21^st^ century, a phenotypical variation increased the efficiency of transmission in *Culex* ssp. mosquitoes^6^. In Brazil, serological indications of viral circulation were detected in various vertebrate species in the Pantanal of Mato Grosso and in the Northeast, warning of the possibility of human cases occurring in the region^17^ ^,^ ^20^. In 2014, the first human case of neuro-invasive disease by WNV in Brazil was serologically confirmed in a resident of the rural area of Piauí state^25^. Viruses such as MAYV, of the *Alphavirus* genus, and the Oropouche virus (OROV), of the *Orthobunyavirus* genus from the *Bunyaviridae* family, have been frequently identified in the Amazonian region in patients with nonspecific feverish conditions or with neurological damage^7^ ^,^ ^15^ ^,^ ^16^. Other arboviruses have been isolated in humans in Brazil, such as Saint Louis encephalitis, in a dengue-suspect case in the state of São Paulo^19^, Ilhéus, Rocio^14^, and Bussuquara^14^ (*Flavivirus* genus), suggesting possible emergence.

## CLINICAL PRESENTATION OF THE ARBOVIRUSES

The clinical manifestations of infection by arbovirus can vary from light feverish undifferentiated disease to neurological, articular, and hemorrhagic feverish syndromes. Frequently, severe cases are only identified after viral circulation in extensive epidemics, many times reflecting the unpredictable impact on morbidity and mortality, while the occurrence, until now, has been restricted to isolated cases or small outbreaks.

Deaths by CHIKV in epidemics were not known until the epidemic of Reunion Island in the Indian Ocean, where severe neurological cases were registered (encephalitis with deaths and aftereffects) and perinatal transmission with encephalitis and retardation of neuro-psychomotor development in children^9^. Similarly, infection by ZIKV was considered an exanthematous benign disease with light, self-limited symptoms, since few cases were known. The diagnosis was changed, however, during the epidemic of October 2013 to March 2014 in French Polynesia, with 29 thousand estimated cases and the notification of neurological conditions (encephalitis, myelitis, and peripheral paralysis) associated with ZIKV^12^ ^,^ ^18^. In addition, occurrences of Guillain-Barré syndrome multiplied by eight times in that location during the time of the epidemic, suggesting that the virus contributed to the etiology of the cases^12^ ^,^ ^18^. After the emergence of ZIKV in Brazil and the rest of the Americas, the infection was associated with Guillain-Barré syndrome, fatal encephalitis in adults, deaths of fetuses, microcephaly, and other malformations of fetuses, (congenital Zika syndrome).

Dengue, after its re-emergence in the context of major epidemics, hyperendimicity and co-circulation of various serotypes, has also been associated with the rise in severe cases throughout the world. Immunity obtained in previous infections leads to the amplification mediated by ADE (antibody dependent enhancement) antibodies, with high viremia and release of inflammatory markers in reinfections by different serotypes, particularly in the second infections^11^.

Until the emergence of WNV in Romania, with a fatality rate of 10%, the neurological forms were considered rare^22^. Since 1999, in the United States, the rapid dispersion of the virus and association with greater epidemics of encephalitis in the country were notable, with heavy impact on morbidity and mortality. Outbreaks in Europe and the Americas still occur, suggesting the geographic expansion of the disease^10^.

The impact of arboviruses on morbidity and mortality intensifies as the extended epidemics assume a great number of affected individuals, with implications on the health services, mainly in light of the absence of treatment, vaccines, and other effective methods of prevention and control.

On the other hand, environmental pressure has led to the selection of viral lineages that cause more intense viremia and, consequently, higher pathogeny of the disease, as in the case of CHIKV and WNV. These viruses are highly adaptable, and can easily emerge and establish themselves in new geographic areas, suggesting that new and old viruses could potentially reappear. A few arboviruses with higher potential of circulation in Brazil are Saint Louis and West Nile (*Flaviviridae* family), Oropouche (*Bunyaviridae* family), Venezuelan encephalitis virus, and Mayaro (*Togaviridae* family), all of which can be associated with severe cases, especially if extensive epidemics occur. All of them have proven to be capable of transmission via potentially urban arthropods of the *Diptera* order, such as *Culicoides paraensis*, *Aedes* ssp., and *Culex* ssp., which are widely occurring in the country^14^.

## PERSPECTIVES

Arboviruses are a growing public health problem worldwide, mainly due to the potential of dispersion, adaptability to new environments and hosts (vertebrate and invertebrate), possibility of causing extensive epidemics, universal susceptibility, and occurrence of high numbers of severe cases, with neurological, articular, and hemorrhagic damage. The introduction of any arbovirus in an indemnified environment or one with the presence of the vector should never be neglected.

The fight against emerging arboviruses requires wide-ranging policies and interventions involving various sectors of society, not only the health sector.

The definitive establishment of *Aedes* in the Americas was associated with climate change, deforestation, disorganized urbanization, growth of cities, absence of water and basic sanitation, and population displacement. These factors define the paths of the diseases, influenced by the pressure of the viral mutation and genetic adaptations of the viruses to hosts, vectors, and new environments. Despite difficulties regarding socioeconomic and environmental factors, the health sector has responsibilities, such as investments in the prevention, diagnosis, and treatment of infections; for example, in the particularly critical case of pregnant women affected by ZIKV.

The development of vaccines has been a challenge for many study groups in Brazil and worldwide, considering its proven viability for various Flaviviruses.

Investments in the qualification of the actions of epidemiologic, virologic, vectorial, and epizootic surveillance are urgent in the country, especially during times of risks important to public health. International collaboration is essential for the early identification of the entrance of new pathogens in indemnified regions; however, integrated policies and actions are particularly strategic in a country the size of Brazil.

Perplexity regarding the spread of ZIKV and CHIKV and their impact in Brazil were sufficient to establish a state of public health emergency by the Ministry of Health and World Health Organization, almost two years after the entrance of the virus in the country. This condition implied an intense mobilization of resources and articulations among states and municipals to combat viral circulation, which took on huge proportions. Thus, the epidemiologic investigation and suspicion of other arboviruses must be part of the epidemiologic surveillance routines and concerns of national public health to predict new epidemiologic emergences. On the other hand, the efforts are essential to the development and improvement of agile, sensitive diagnostic exams with little cross-reactions with other arboviruses – specific immunobiologicals and synthesis of antiviral medication, mainly due to the infection of pregnant women by ZIKV. Joint actions in research and the fight against the vectors could affect the expansion of emerging viruses, such as infection by CHIKV and ZIKV, the greatest concerns in the country at the moment.

## Footnotes

a Pan American Health Organization. Dengue. Washington (DC); s.d [cited 2015 Dec 22]. Available from: http://www.paho.org/hq/index.php?option=com_topics&view=article&id=1&Itemid=40734
